# The impact of Raynaud’s phenomenon on work ability – a longitudinal study

**DOI:** 10.1186/s12995-022-00354-2

**Published:** 2022-06-08

**Authors:** Albin Stjernbrandt, Jens Wahlström

**Affiliations:** Department of Public Health and Clinical Medicine, Section of Sustainable Health, Umeå University, 901 87 Umeå, Sweden

**Keywords:** Raynaud Disease, Work, Sick Leave, Longitudinal Studies, Sweden

## Abstract

**Objective:**

To determine if having Raynaud’s phenomenon (RP) affects the work ability, job retainment, or occurrence of sick leave.

**Methods:**

Surveys on the working-age general population of northern Sweden were conducted in 2015 and 2021, gathering data on RP, occupation and sick leave. Work ability was assessed using the Work Ability Score.

**Results:**

The study population consisted of 2,703 women and 2,314 men, among which 390 women and 290 men reported RP at follow-up. For women, the mean [standard deviation (SD)] Work Ability Score was 8.02 (2.24) for subjects reporting RP and 7.68 (2.46) for those without RP. For men, the corresponding numbers were 7.37 (2.03) and 7.61 (2.14), respectively. Multiple linear regression did not show an association between RP status and work ability (*p* = 0.459 for women and *p* = 0.254 for men), after adjusting for age, body mass index, physical workload, cardiovascular disease, and perceived stress. Having retained the same main livelihood since baseline was reported by 227 (58.5%) women with RP, 1,163 (51.2%) women without RP, 152 (52.6%) men with RP, and 1,075 (54.1%) men without RP (*p* = 0.002 for women and *p* = 0.127 for men). At follow-up, any occurrence of sick leave during the last year was reported by 80 (21.4%) women with RP, 410 (18.6%) women without RP, 48 (17.1%) men with RP, and 268 (13.7%) men without RP (*p* = 0.208 for women and *p* = 0.133 for men). Among those reporting sick leave, the mean (SD) duration in months was 2.93 (3.76) for women with RP, 3.00 (4.64) for women without RP, 2.77 (3.79) for men with RP, and 2.91 (12.45) for men without RP (*p* = 0.849 for women and *p* = 0.367 for men).

**Conclusion:**

For neither women nor men was there a significant effect of having RP on work ability. Women with RP reported a slightly higher job retainment compared to those without the condition, while there was no difference in job retainment among men. For neither gender did the presence of RP influence the occurrence of recent sick leave, nor did it affect the length of time away from work.

**Supplementary Information:**

The online version contains supplementary material available at 10.1186/s12995-022-00354-2.

## Introduction

Raynaud’s phenomenon (RP) is the clinical manifestation of vasospasm affecting digital blood vessels [1]. It can be defined as episodes of peripheral blanching of the fingers, triggered by exposure to cold, vibration, or psychological stress [2]. RP is a common condition, with prevalence figures of around 12–14% in the Scandinavian general population [3, 4]. As often caused by occupational exposure to hand-transmitted vibration, Swedish insurance statistics from 2018 report that RP is the most commonly compensated occupational injury, representing over one third of all approved claims [5]. In current practice, subjects with RP are classified either as primary RP, when no underlying condition is found, or secondary RP, when there is associated disease. Suffering from RP can have a major effect on the quality of life [6, 7]. However, little is known about impacts on work ability, job retainment, or sick leave.

The concept of work ability is complex and entails the balance between physical and cognitive demands in relation to the resources of the individual, modified by the organizational context [8]. In addition, both work demands and individual resources are dynamic factors that change over time [9]. It could be postulated that suffering from RP should mainly be a hindrance for subjects performing manual outdoor work, since exposure to cold climate triggers vasospastic attacks [10], and RP decreases manual dexterity and physical performance [4, 11]. However, as mentioned above, psychological stress is also an established trigger for RP attacks and should be considered in this context. One method for measuring work ability is the Work Ability Score (WAS) from the Work Ability Index (WAI) questionnaire, which is a commonly used and well-validated tool [8]. The WAS consists of a whole number numerical rating scale ranging from 0–10, where the current work ability is subjectively compared to the lifetime best. The WAS has been shown to have an equally good predictive value as the whole WAI instrument regarding health, pain, and sick leave [12, 13]. Previous studies on Swedish workers have shown that age and psychological mood have significant impacts on the WAS [9, 14]. External factors that can affect work ability include ambient temperature, humidity, dust and noise levels, hand-arm vibration, and ergonomic exposures [15, 16]. However, the effects of such physical work factors are modified by the health status of the individual worker. For instance, it is likely that exposure to ambient cold and vibration poses a greater challenge to a subject with RP than a completely healthy individual. In this context, a few studies have investigated effects on work ability in specific groups of patients with secondary forms of RP, such as vibration-induced white fingers [14, 16, 17] and systemic sclerosis [18]. However, to the authors’ knowledge, there are no previous population-based studies on the matter.

The primary aim of this study was to determine if having Raynaud’s phenomenon affects the work ability, job retainment, or occurrence of sick leave. Secondary aims were to investigate longitudinal effects of incident or remittent Raynaud’s phenomenon on work ability, and evaluate potential gender differences.

## Methods

### Study design and setting

This prospective closed-cohort study was part of the Cold and Health In Northern Sweden (CHINS) research project, which was initiated in 2015 to broadly explore adverse health effects from ambient cold exposure, and has previously been described in detail [3]. The study sample included men and women of working age at enrollment (18–70 years), living in northern Sweden, who were recruited from the national Swedish population register. Baseline data came from the first postal survey that was administered between February and May of 2015. Follow-up data was retrieved through a digital questionnaire that collected data between March and April of 2021. All subjects who had responded to the baseline questionnaire were invited by a postal query to respond to the follow-up questionnaire, with one postal reminder. Subjects who were unable to answer digitally were given the option to respond to the questionnaire on paper.

### Variables and statistical analyses

Responses from both surveys were merged based on social security numbers. Continuous variables data were described as mean values with standard deviation (SD), while categorical variables were presented as numbers and valid percentages. Subjects were defined as having RP through a positive response to a single questionnaire item that was present in both surveys: “Does one or more of your fingers turn white (as shown on picture) when exposed to moisture or cold?”, and this was supported by a previously developed color chart [19]. In the follow-up survey, study participants were also asked additional questions about year of first occurrence, attack frequency and distribution, as well as progression of RP. Length and weight were collected at baseline, in order to calculate body mass index (BMI). Work ability was assessed using the WAS, which was included in the follow-up survey. Current occupation was specified in free-form text in both surveys, and manually coded in accordance with the two-level International Standard Classification of Occupations (ISCO) [20]. Physical workload was determined by a previously published job–exposure matrix (JEM) that categorized the exposure into low (e.g. desk jobs), medium (e.g. ambulatory work) or high (e.g. heavy lifting or climbing), based on the ISCO coding at baseline [21]. Perceived stress was asked about in both surveys, and responses dichotomized so that “none/very little/some” was considered a negative response, and “quite a lot/very much” a positive response. Occupational exposure to outdoor or cold environments at baseline and follow-up were reported on a ten-level whole number numerical rating scale, ranging from “do not agree” to “fully agree”, and responses dichotomized based on the 50^th^ percentile. Cardiovascular diseases were asked about in the baseline survey, and included the presence of physician-diagnosed hypertension, angina pectoris, myocardial infarction, or stroke. The Mann–Whitney U test and Pearson’s chi square test was used to determine statistical differences for continuous and categorical variables, respectively. Simple and multiple linear regression was used to model the relations between the WAS and independent variables (i.e. RP status, age, BMI, physical workload, cardiovascular disease, and perceived stress). Statistical tests were chosen based on the distribution of data, and non-parametric tests opted for when the assumption of normal distribution was violated. A *p* value < 0.05 was considered statistically significant. Statistical analyses were performed using SPSS (version 27.0, IBM Corporation, Armonk, NY, USA).

## Results

### Recruitment

**Fig. 1:**
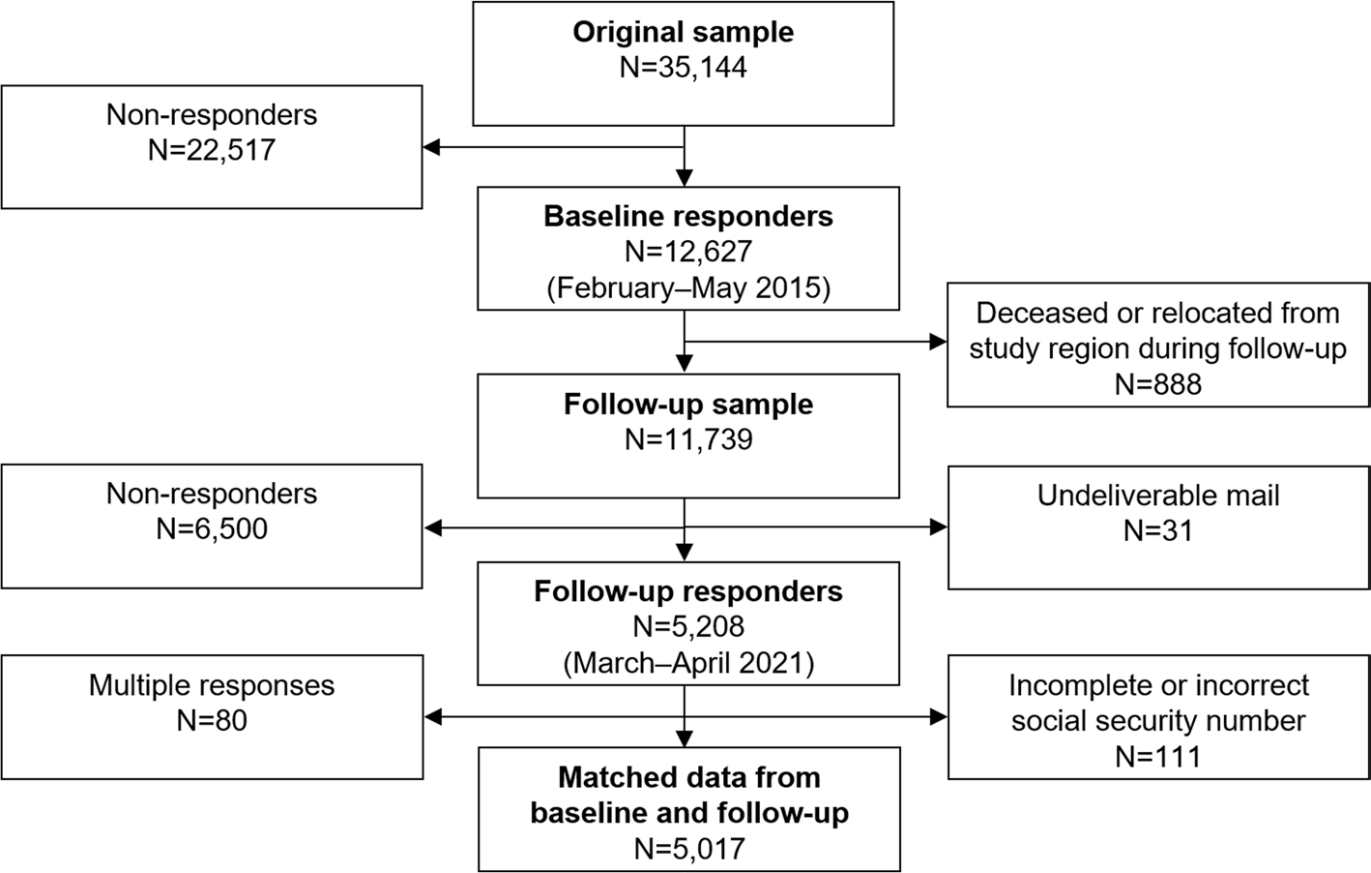
Participation tree showing the data collection for the baseline and follow-up surveys

The baseline cohort (2015) consisted of 12,627 subjects, of which 888 were deceased or had moved from the study region at the time of follow-up (2021). For an additional 31 subjects, the written invitation to participate in the follow-up survey could not be delivered by the postal services. There were 5,208 responses to the follow-up survey, yielding a response rate of 44.4%. Due to multiple responses (*N* = 80) and invalid social security numbers (*N* = 111), all survey responses could not be matched to the original dataset, leaving 5,017 subjects available for analysis (Fig. 1). Five subjects (0.1%) requested a postal follow-up survey. Details on sampling and response rates are presented in Additional file 1.

### Characteristics of the study population

The final study population consisted of 2,703 women (53.9%) and 2,314 men. Other baseline characteristics are presented in Table 1. There were 390 women (14.5%) and 290 men (12.7%) reporting RP affecting the hands at follow-up. Among these subjects, the mean (SD) age of onset of RP was 31 (15) years for women and 36 (17) years for men. Having had vasospastic episodes during the last two years was reported by 328 women (88.6%) and 230 men (85.1%). Regarding distribution of blanching, 330 women (85.1%) and 240 men (83.7%) reported affection of middle and/or distal phalanges for the right hand, while the corresponding figures for the left hand was 338 (85.1%) and 237 (83.7%), respectively. An increased attack frequency since onset was reported by 93 women (24.1%) and 103 men (35.8%), while an increased distribution of blanching was reported by 50 (13.0%) and 47 (12.3%), respectively.

**Table 1:** Baseline characteristics of the study participants

Baseline variable	Women				Men			
	**RP + **		**RP − **		**RP + **		**RP − **	
	N (%)	Mean (SD)	N (%)	Mean (SD)	N (%)	Mean (SD)	N (%)	Mean (SD)
Study participants	390 (14.5)		2,291 (85.5)		290 (12.7)		1,994 (87.3)	
Age (at enrollment)		50 (12)		51 (13)		55 (12)		53 (13)
Body mass index (kg/m^2^)		23.7 (3.7)		26.0 (5.1)		25.6 (3.4)		26.7 (3.9)
Current daily smoking	14 (3.6)		170 (7.5)		14 (4.8)		86 (4.4)	
Cigarettes per day		10 (5)		9 (5)		11 (6)		11 (7)
Current daily snuff use	33 (8.5)		161 (7.1)		80 (27.8)		383 (19.4)	
Snuff boxes per week		4 (3)		4 (3)		4 (2)		4 (3)
Occupation ^a^								
Armed forces	0 (0.0)		1 (0.1)		3 (1.1)		14 (0.7)	
Managers	27 (7.1)		105 (4.7)		22 (7.8)		105 (5.4)	
Professionals	118 (31.1)		629 (27.9)		41 (14.5)		298 (15.3)	
Technicians and associate professionals	42 (11.1)		211 (9.4)		46 (16.3)		291 (15.0)	
Clerical support workers	44 (11.6)		269 (12.0)		14 (4.9)		146 (7.5)	
Service and sales workers	49 (12.9)		402 (17.9)		14 (4.9)		142 (7.3)	
Skilled agricultural, forestry and fishery workers	2 (0.5)		15 (0.7)		3 (1.1)		41 (2.1)	
Crafts and related trades workers	5 (1.3)		26 (1.2)		30 (10.6)		177 (9.1)	
Plant and machine operators and assemblers	7 (1.8)		31 (1.4)		38 (13.4)		214 (11.0)	
Elementary occupations	8 (2.1)		44 (2.0)		3 (1.1)		43 (2.2)	
Self-employed	6 (1.6)		36 (1.6)		6 (2.1)		51 (2.6)	
Sick leave	9 (2.4)		32 (1.4)		1 (0.4)		11 (0.6)	
Parental leave	2 (0.5)		20 (0.9)		0 (0)		0 (0)	
Students	14 (3.7)		84 (3.7)		3 (1.1)		53 (2.7)	
Retired	42 (11.1)		312 (13.9)		56 (19.8)		324 (16.7)	
Physical workload ^b^								
Low	243 (75.5)		1,289 (69.6)		124 (55.6)		875 (55.6)	
Medium	31 (9.6)		201 (8.8)		51 (22.9)		388 (24.6)	
High	48 (14.9)		363 (19.6)		48 (21.5)		312 (19.8)	
Occupational cold exposure ^c^								
Low	255 (67.8)		1,552 (70.3)		115 (42.3)		905 (47.1)	
High	121 (32.2)		655 (29.7)		157 (57.7)		1,017 (52.9)	
Cardiovascular disease ^d^								
No	323 (85.4)		1,762 (78.4)		213 (76.3)		1,383 (71.7)	
Yes	55 (14.6)		485 (21.6)		66 (23.7)		545 (28.3)	

Baseline variables have been stratified by gender and the presence of Raynaud’s phenomenon at follow-up

*RP* Raynaud’s phenomenon

^a^ According to the International Standard Classification of Occupations (ISCO) 2008

^b^ According to the job-exposure matrix

^c^ Self-reported occupational exposure to outdoor or cold environments

^d^ Physician-diagnosed hypertension, angina pectoris, myocardial infarction, or stroke

### Work ability

Among women, the mean (SD) WAS was 8.02 (2.24) for subjects reporting RP at follow-up and 7.68 (2.46) for those without RP. For men, the corresponding numbers were 7.37 (2.03) and 7.61 (2.14), respectively. In unadjusted analyses, there was a significant effect of RP status on the WAS among women, but not men (Table 2). However, in adjusted analyses, also including age, BMI, physical workload, cardiovascular disease, and perceived stress, there was no significant effect of RP status on the WAS for either gender (Table 3).

**Table 2:** Simple linear regression for the Work Ability Score

		**Unstandardized coefficients**			**Standardized coefficients**			
			**95% CI**					
**Gender**	**Variable**	**B**	**Lower**	**Upper**	**Beta**	**t-ratio**	***p* value**	**R**^**2**^
Women	Raynaud’s phenomenon (yes versus no)	0.45	0.07	0.62	0.05	2.49	0.013	0.002
	Age (years)	− 0.07	− 0.08	− 0.07	− 0.38	− 20.66	< 0.001	0.147
	Body mass index (kg/m^2^)	− 0.07	− 0.09	− 0.06	− 0.15	− 7.57	< 0.001	0.023
	Physical workload (high or medium versus low)	− 0.59	− 0.79	− 0.38	− 0.12	− 5.63	< 0.001	0.015
	Cardiovascular disease (yes versus no)	− 1.53	− 1.77	− 1.29	− 0.29	− 12.53	< 0.001	0.061
	Perceived stress (high versus low)	− 0.48	− 0.70	− 0.26	− 0.09	− 4.24	< 0.001	0.007
Men	Raynaud’s phenomenon (yes versus no)	− 0.24	− 0.51	0.03	− 0.04	− 1.75	0.080	0.001
	Age (years)	− 0.07	− 0.08	− 0.07	− 0.45	− 23.26	< 0.001	0.198
	Body mass index (kg/m^2^)	− 0.05	− 0.07	− 0.03	− 0.09	− 4.05	< 0.001	0.007
	Physical workload (high or medium versus low)	− 0.62	− 0.80	− 0.43	− 0.16	− 6.66	< 0.001	0.024
	Cardiovascular disease (yes versus no)	− 1.22	− 1.42	− 1.02	− 0.25	− 12.01	< 0.001	0.064
	Perceived stress (high versus low)	0.04	− 0.22	0.30	0.01	0.28	0.777	< 0.001

All variables have been analyzed separately, and results stratified by gender. *B* unstandardized coefficients, *95% CI* ninety − five percent confidence interval for the unstandardized coefficients, *Beta* standardized coefficients, *R*.^*2*^ adjusted coefficient of determination

**Table 3:** Multiple linear regression for the Work Ability Score

		**Unstandardized coefficients**			**Standardized coefficients**			
			**95% CI**					
**Gender**	**Variable**	**B**	**Lower**	**Upper**	**Beta**	**t-ratio**	***p* value**	**R**^**2**^
Women	Raynaud’s phenomenon (yes versus no)	0.10	− 0.16	0.35	0.02	0.74	0.459	
	Age (years)	− 0.05	− 0.06	− 0.04	− 0.26	− 11.93	< 0.001	
	Body mass index (kg/m^2^)	− 0.02	− 0.04	− 0.01	− 0.05	− 2.26	0.024	
	Physical workload (high or medium versus low)	− 0.47	− 0.67	− 0.27	− 0.10	− 4.70	< 0.001	
	Cardiovascular disease (yes versus no)	− 0.55	− 0.81	− 0.30	− 0.09	− 4.19	< 0.001	
	Perceived stress (high versus low)	− 0.54	− 0.74	− 0.34	− 0.11	− 5.27	< 0.001	
	Explained variance proportion for full model							0.12
Men	Raynaud’s phenomenon (yes versus no)	− 0.15	− 0.41	0.11	− 0.03	− 1.14	0.254	
	Age (years)	− 0.05	− 0.06	− 0.04	− 0.32	− 13.42	< 0.001	
	Body mass index (kg/m^2^)	− 0.01	− 0.03	0.02	− 0.02	− 0.63	0.530	
	Physical workload (high or medium versus low)	− 0.51	− 0.67	− 0.33	− 0.13	− 5.75	< 0.001	
	Cardiovascular disease (yes versus no)	− 0.45	− 0.67	− 0.22	− 0.10	− 3.95	< 0.001	
	Perceived stress (high versus low)	− 0.34	− 0.58	− 0.11	− 0.07	− 2.88	0.004	
	Explained variance proportion for full model							0.16

All variables have been included simultaneously, and results stratified by gender. *B* unstandardized coefficients, *95% CI* ninety − five percent confidence interval for the unstandardized coefficients, *Beta* standardized coefficients, *R*.^*2*^ adjusted coefficient of determination

In longitudinal analyses, there were 96 women and 112 men who negated RP at baseline but reported RP during follow-up. For women, the mean (SD) WAS was 8.18 (2.19) among such incident cases and 7.71 (2.44) among subjects who negated RP both at baseline and follow-up (*p* = 0.077). The corresponding figures for men were 7.23 (2.11) and 7.63 (2.14) (*p* = 0.025). In addition, there were 104 women and 88 men that reported RP at baseline but remitted during follow-up. For women, the mean (SD) WAS was 7.09 (2.93) among remitted cases and 7.94 (2.26) among those with persistent RP (*p* = 0.032). The corresponding figures for men were 7.09 (2.25) and 7.50 (1.94) (*p* = 0.239).

### Job retainment and sick leave

**Fig. 2:**
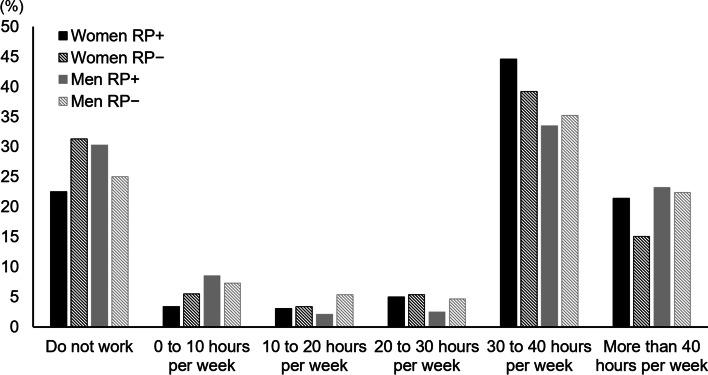
Reported working time for subjects with and without Raynaud’s phenomenon

Having retained the same main livelihood since the baseline survey (2015) was reported by 227 (58.5%) women with RP, 1,163 (51.2%) women without RP, 152 (52.6%) men with RP, and 1,075 (54.1%) men without RP (*p* = 0.002 for women and *p* = 0.127 for men). Reasons for changing livelihood are presented in Table 4. Among those who had changed occupation during follow-up, no transition from outdoor to indoor work could be discerned, due to a small and heterogenous material. Access to occupational health care at follow-up was reported by 199 (51.8%) women with RP, 1,012 (45.3%) women without RP, 125 (43.9%) men with RP, and 886 (44.9%) men without RP (*p* = 0.004 for women and *p* = 0.407 for men). Among currently working subjects, a high number of weekly work hours was reported by women with RP (Fig. 2). At follow-up (2021), any occurrence of sick leave during the last twelve months was reported by 80 (21.4%) women with RP, 410 (18.6%) women without RP, 48 (17.1%) men with RP, and 268 (13.7%) men without RP (*p* = 0.208 for women and *p* = 0.133 for men). Among those reporting sick leave, the mean (SD) duration in months was 2.93 (3.76) for women with RP, 3.00 (4.64) for women without RP, 2.77 (3.79) for men with RP, and 2.91 (12.45) for men without RP (*p* = 0.849 for women and *p* = 0.367 for men).

**Table 4:** Reason for change of main livelihood between 2015–2021

	**Women**		**Men**	
	**RP + **	**RP-**	**RP + **	**RP-**
	N (%)	N (%)	N (%)	N (%)
Change of main livelihood	89	738	100	593
Retired	70 (78.7)	647 (87.7)	97 (97.0)	557 (93.9)
Job change	5 (5.6)	22 (3.0)	0 (0)	13 (2.2)
Sick leave	4 (4.5)	15 (2.0)	1 (1.0)	2 (0.3)
Disability pension	7 (7.9)	28 (3.8)	0 (0)	11 (1.9)
Further education	2 (2.2)	14 (1.9)	2 (2.0)	10 (1.7)
Parental leave	1 (1.1)	12 (1.6)	0 (0)	0 (0)

Data have been stratified by gender and the presence of Raynaud’s phenomenon at follow-up

*RP* Raynaud’s phenomenon

## Discussion

### Main findings

Our study did not reveal a significant effect of having Raynaud’s phenomenon on work ability, when analyzed using multiple linear regression. Women with Raynaud’s phenomenon reported a slightly higher job retainment compared to those without the condition, and generally long working hours. There were no statistically significant differences in sick leave occurrence or duration.

### Interpretation and comparison with other studies

The prevalence of RP in the present surveys was comparable with the roughly 12% that was reported in a Finnish population-based study [4]. The condition was more common among women, which is also in line with previous research [10]. Further, the mean age of onset of 31 years for women and 36 years for men was quite similar to the results of a meta-analysis on longitudinal studies on RP (10 studies; 639 subjects), where the mean age of onset was 34 years [22].

The present study did not show any significant effect of RP status on work ability in the multiple linear regression models, when also using age, BMI, physical workload, cardiovascular disease, and perceived stress as covariates. However, in unadjusted analyses, there was a significant positive effect of RP status on work ability among women. Also, the results of longitudinal analyses suggested that men with incident RP had a slightly lower work ability than healthy subjects, while women with remitted RP reported a lower work ability than those with persistent disease. It is plausible that RP is indeed a hindrance for work, especially in manual outdoor occupations where exposure to ambient and contact cold, as well as hand-arm vibration, can trigger vasospastic attacks. This might reduce the work capacity in tasks requiring grip force and manual dexterity, and motivate the worker to seek a heated environment in order to regain full use of the hands. Such manual outdoor occupations are common among working men in northern Sweden, as evidenced both by the descriptive analyses on occupation in this study (Table 1), as well as official statistics from the Swedish Work Environment Authority [23]. In contrast, women with RP reported long working hours and a higher job retainment than their healthy counterparts. These finding are harder to explain, but may be due to the fact that RP does not pose a hindrance for indoor work with low physical demands, that were common among women in the study population. It is also possible that the work participation facilitation was more efficient for women with RP, since they reported a higher access to occupational health care. As shown in Table 4, the large majority of those who changed the main livelihood had retired, and only a few percent had changed into another field of work. This is at least in part explained by the age composition of the study sample, in which a large proportion had reached the general retirement age of 65 years by the time of follow-up. Among subjects with RP who had changed field of work, a distinct transition from outdoor to indoor tasks during the follow-up time could not be discerned. However, this subgroup contained few responding subjects and revealed a large variation regarding new occupations, and these facts limit what conclusions could be drawn.

Importantly, most subjects reported a good work ability, regardless of having RP or not. Neither were there any significant effects on sick leave parameters. In this context, it is important to recall that most subjects reported a mild state of RP, regarding both attack frequency, distribution of paleness, and disease progression over time. Thus, it is reasonable to assume that the condition only had a minor impact on work ability. However, concern has been raised if the WAS sufficiently captures limitations in work ability for conditions that only affect the hands [14], since it only gives a rough measure of the global work ability. A more specific item for measuring hand disability, such as the hand disability index of the Stanford Health Assessment Questionnaire [24], might have revealed larger differences between groups. Also, since the WAS relates the current work ability to the lifetime best, there is a risk that perceived effects on work ability are attenuated among subjects with long-standing conditions, such as RP.

Regarding the effects of other factors on work ability, the present study showed a significant effect of age on the WAS. A previous Swedish study on work ability among vibration-exposed workers subjects, where the prevalence of RP was 30% among men and 50% among women, reported an effect of age and distribution of neurosensory symptoms, but not vascular symptoms [16]. The β coefficient for age ranged from − 0.07 to − 0.09, closely resembling the results in the present study. Our study demonstrated a negative impact of high BMI on the WAS, however in the adjusted analyses only statistically significant among women. A high BMI has also previously been associated to poorer work ability, most likely due to reduced physical capacity, although overweight could also be a proxy marker for other disease [12]. In the present study, high perceived stress negatively affected work ability, with a stronger association among women. This is in line with previous research that has shown associations between stress levels and work ability, as well a greater susceptibility for stress among women [12, 13].

### Limitations

There was a large proportion of survey non-responders, and an underrepresentation of younger age groups among responders (as presented in Additional file 1) that could have affected the generalizability of the results and introduced a sampling bias. Although socioeconomic status may affect work ability, the present study collected no data on such parameters, other than occupational title. The validity of the diagnosis of RP can be questioned, since it was based on a single questionnaire item, although supported by a previously developed color chart that has previously been shown to increase both sensitivity and specificity in comparison with only posing questions [19, 25]. Using more specific criteria, or performing a thorough examination by a physician, would likely have increased the diagnostic accuracy. However, such clinical investigation was not feasible due to the large study size. Furthermore, the surveys were not designed to separate between primary and secondary RP, and it is plausible that secondary RP might negatively affect work ability more so than primary RP. Also, the underlying conditions of patients with diseases giving rise to secondary RP could have had a larger impact on work ability than the symptoms of RP in itself. Thus, in future studies on work ability in this context, more attention should be given to the etiology of RP. Finally, the low explained variance proportions of the multiple linear regression models suggest that there are other important factors that affect work ability that were not investigated in our study.

### Strengths

To the authors’ knowledge, this is the first population-based prospective study on work ability among subjects with RP. The study was performed in a Scandinavian setting, where the condition is quite common. The surveys were detailed and enabled longitudinal analyses with six years of follow-up time. Also, both surveys were distributed during the same late-winter season, so that cold exposure, which often serves as a trigger for vasospastic symptoms, would be comparable. Also, since previous studies have reported on differences in RP between women and men [10, 26], all analyses were stratified by gender.

## Conclusions

For neither women nor men was there a significant effect of having Raynaud’s phenomenon on work ability. Women with Raynaud’s phenomenon reported a slightly higher job retainment compared to those without the condition, while there was no difference in job retainment among men. For neither gender did the presence of Raynaud’s phenomenon influence the occurrence of recent sick leave, nor did it affect the length of time away from work. These results suggest that having Raynaud’s phenomenon does not have a profound effect on the participation in working life.

## Supplementary Information

**Additional file 1. **Distribution of age, gender, and county for the sampling frame, baseline survey responders, and follow-up survey responders. Analysis of responder patterns for the baseline and follow-up survey.

## Data Availability

The dataset supporting the conclusions of this article can be made available on personal request to the corresponding author.

## Abbreviations

B: Unstandardized coefficients
Beta: Standardized coefficients
BMI: Body mass index
CHINS: Cold and Health In Northern Sweden
ISCO: International Standard Classification of Occupations
JEM: Job–exposure matrix
RP: Raynaud’s phenomenon
R^2^: Adjusted coefficient of determination
SD: Standard deviation
WAI: Work Ability Index
WAS: Work Ability Score
95% CI: Ninety − five percent confidence interval
