# Supplementary material for: The impact of Raynaud’s phenomenon on work ability – a longitudinal study
Source: J Occup Med Toxicol. 2022 Jun 8;17:12. doi: 10.1186/s12995-022-00354-2 (PMC9175314; doi:10.1186/s12995-022-00354-2)
Supplement: Supplementary file 1 — Additional file 1. Distribution of age, gender, and county for the sampling frame, baseline survey responders, and follow-up survey responders. Analysis of responder patterns for the baseline and follow-up survey. [file 12995_2022_354_MOESM1_ESM.pdf]

Additional file 1: Distribution of age, gender, and county for the sampling frame, baseline survey responders, and follow-up survey responders.

Article title: The impact of Raynaud's phenomenon on work ability – a longitudinal study

Journal name: Journal of Occupational Medicine and Toxicology

Corresponding author: Albin Stjernbrandt, Section of Sustainable Health, Department of Public Health and Clinical Medicine, Umeå University, albin.stjernbrandt@umu.se

	Sampling frame		Baseline survey responders		Follow-up survey responders	
**Variable**	N	%	N	%	N	%
**Age group (years)** [a]						
18–31	9,328	26.5	1,816	14.4	476	9.5
32–44	7,553	21.5	2,167	17.2	872	17.4
45–57	8,894	25.3	3,486	27.6	1,603	32.0
58–70	9,369	26.7	5,158	40.8	2,066	41.2
**Gender**						
Women	17,589	50.0	6,886	54.5	2,703	53.9
Men	17,555	50.0	5,741	45.5	2,314	46.1
**County**						
Norrbotten	9,036	25.7	3,115	24.7	1,179	23.5
Västerbotten	10,627	30.2	3,944	31.2	1,710	34.1
Västernorrland	8,905	25.3	3,203	25.4	1,202	24.0
Jämtland	6,576	18.7	2,365	18.7	926	18.5

[a] Age at enrollment (2015).
